# The Challenges of Conducting a Nurse-Led Intervention in a Randomized Controlled Trial with Vulnerable Participants

**DOI:** 10.1155/2014/394237

**Published:** 2014-04-30

**Authors:** Tanya Park, Kim Usher, Kim Foster

**Affiliations:** ^1^School of Nursing, Midwifery and Nutrition, James Cook University, P.O. Box 6811, Cairns, QLD 4870, Australia; ^2^School of Health, University of New England, Armidale, NSW 2350, Australia; ^3^Faculty of Health, Disciplines of Nursing & Midwifery, University of Canberra, Canberra, ACT 2600, Australia

## Abstract

This paper discusses the challenges encountered by researchers while conducting a randomized controlled trial (RCT) testing the efficacy of a healthy lifestyle educational and exercise intervention for people with serious mental illness. RCTs, even though considered the “gold standard” of research designs, are still prone to risks of potential bias and threats to their validity. Based on researcher reflexivity, the combination of reflection and action, during the conduct of the study, this paper outlines a number of challenges faced by the researchers. These included managing the need of participants to tell their story and be heard, reluctance of participants to remain in allocated groups, participant literacy, dual role of the nurse nurse-researcher, and reporting the benefits of nonstatistical results of a quantitative research project. Recommendations for conducting future behaviour intervention studies of this type include the incorporation of a reflexive component for the nurse nurse-researcher, highlighting the importance of taking a reflexive stance in both qualitative and quantitative research designs.

## 1. Introduction

*“It's all about the numbers and statistics; it's not meant to be about the stories. Isn't that what qualitative researchers do?”*

This paper critically discusses challenges encountered by researchers in delivering a nurse-led healthy lifestyle intervention for people with a serious mental illness (SMI) prescribed second-generation antipsychotic medications. A randomised controlled trial (RCT) was conducted to test the effectiveness of the intervention on individuals' weight measurements including girth and Body Mass Index (BMI). This experimental design study tested the relationship between the delivery of a specifically designed nurse-led intervention, “Passport 4 Life,” and weight gain prevention or weight maintenance in people with serious mental illness taking SGAs. The aim of the study was to determine whether a nurse-led intervention can have an impact on weight maintenance or reduction or ameliorate weight gain for people with serious mental illness (SMI) already prescribed and taking SGAs. See [1] for further detail on the study.

RCTs, which have superior status amongst experimental design studies and are often referred to as the “gold standard” of research methods [2–4], are considered the most appropriate way to evaluate the impact of such an intervention in clinical practice [5]. The reputation of rigorously conducted RCTs enables the findings of a well-developed and applied study to be considered high level evidence on which future practice in healthcare is based [3, 6]. However, RCTs involving complex psychosocial interventions can be challenging to implement with vulnerable participants, including those with a mental illness such as schizophrenia [7]. This paper discusses the practical and emotional challenges faced by the researchers when conducting a RCT in the real world of clinical practice with this potentially vulnerable population group. Our findings may be useful for other researchers preparing to conduct behavioural intervention studies with participants with mental illness.

## 2. The Randomized Controlled Trial Design

A randomised controlled trial (RCT) is undertaken to test a cause and effect relationship between an intervention and participant outcomes. The intervention tested in this study was a healthy lifestyle program and the effects were measured by collecting weight-related data, including girth measurement and BMI. The rigour and reputation of RCT designs enable the outcome of the findings to be applied from the sample population to the whole population. Statistical results of a RCT enable universal explanation of the sample population, and therefore generalisations, of observable phenomena. Generalisations are a fundamental tenet of this paradigm and allow explanations to be developed with validated statistical consistency [4]. The methodological reputation of RCTs enables the findings of a RCT to be considered quality evidence on which healthcare is based, with some authors suggesting that RCTs are the best way to answer questions of healthcare effectiveness [2, 3, 6].

There are three essential design elements of RCTs: randomisation, control, and manipulation. Randomisation ensures that each study participant has an equal chance of being allocated to the intervention or control group [8]. In the current study, participants were randomly allocated to the control or intervention group. Random allocation occurred after explanation to participants and via the use of opaque envelopes that were selected by participants; each envelope allocated the participant to one of the groups, enabling the participants variables to all have an equal opportunity to be randomly allocated and controlling for potential confounding bias. Random allocation of participants allows the researcher to attribute the study finds to the intervention tested.

The control elements of RCTs enable the researcher to control for potential bias by the use of inclusion/exclusion criteria, concealment during the randomisation process, and blinding [4]. In this study, two of the three control elements, concealment of randomisation and inclusion/exclusion criteria, were used. Blinding participants was not possible. Blinding is not always possible in intervention studies where it is difficult to blind the participant to the intervention that they are or are not participating in [4]. Allocation concealment is a method that can be used when blinding is not possible and, as Schulz and Grimes [9] report,* conveys a strong message of bias prevention. *In this study, participants were required to attend weekly groups if they were allocated to the intervention group and therefore it was not possible to blind participants to the intervention, but it was possible to conceal the intervention during random allocation.

## 3. Challenges Encountered during the RCT

The researchers conducted a RCT to test the effect of a 12-week healthy lifestyle program on participants' weight and girth measurements; the program was designed specifically for people with serious mental illness who had gained weight while taking second generation antipsychotic medications affecting participants' weight and girth measurements (see [1] for an overview of the intervention). The healthy lifestyle intervention program was manipulated for the control and intervention group. The intervention group participated in weekly group sessions that included lifestyle education, healthy snack examples, and 30 minutes of exercise, while the control group were given the educational material in written form only. The results, although not statistically significant, did demonstrate a trend towards weight reduction in the intervention group (see [10] for the study results).

Clinical intervention research, such as RCTs, is often of relatively short duration in comparison to the length of chronic disorders such as schizophrenia. Positive results of intervention studies such as the healthy lifestyle program tested in this study can take an extended period of time to occur. Similar intervention studies to the current study have reported similar results with no statistical significance [11–15]. RCTs are measured by statistical analysis; however, statistical results alone do not necessarily reveal all the outcomes of a study. The challenges specific to conducting this RCT and the lessons learned included the need to allow time for participants' stories during weekly intervention sessions, issues with randomisation, individual exercise challenges, literacy, and the dual identity of the nurse nurse-researcher. These are critically discussed in the following section.

### 3.1. The Need for Time and Space for Participants' Stories in the Weekly Educational Sessions

Very early in the weekly lifestyle and education intervention, it became evident that we had underestimated participants' needs to share their experiences and the importance of listening to participants' stories of weight gain associated with the antipsychotic medications. Working closely with the participants and hearing their stories were a constant reminder of the significant negative impact the weight gain had on their lives. Hearing these stories was difficult for the research team as they conveyed participants' feelings of hopelessness, helplessness, stigmatisation, and despair in relation to their weight gain and their mental illness. A RCT design focuses on the rigorous conduct of an intervention rather than the human aspects of experience. As such, we had not anticipated the need to incorporate time for personal sharing in the weekly sessions. While we had not originally planned for them, we quickly realised the importance of these stories and the need to include story time in the weekly sessions. One outcome of this unanticipated finding was the development of a qualitative project to study the impact of weight gain on mental health consumers while prescribed and taking second generation antipsychotic medications (see [10] for the findings of this study).

### 3.2. The Problem of Randomisation

A further issue that arose in the study was randomisation. Randomisation is recognised as problematic in clinical trials. In fact, participant resistance to random allocation to treatment groups has led to the development and use of preference trial designs where the participant can opt for a preferred treatment before commencement of the RCT. This approach results in a cohort study embedded within a RCT [16]. In the current study, participants were randomly allocated to the control or intervention group. The process of random allocation was explained to participants prior to entering the study. Despite indicating their understanding of this part of the research process prior to study commencement, some of the participants allocated to the control group attended the weekly intervention group sessions despite being allocated to the control group. Even though their allocation to the other group was explained again, they were reluctant to leave the group even when informed that they could join it at a later stage. To manage this issue, the research team had to utilise the process of intention to treat analysis. Intention to treat analysis is the process of analysing data according to randomisation to minimise bias and data loss [17]. The data collected was analysed in the groups that participants were randomly allocated to, despite some of the participants not remaining in their original group (control or intervention) [17].

### 3.3. Participant Concerns about the Exercise Component of the Intervention

Exercise is an essential component of healthy lifestyle change and the Australian Guidelines for Adults [18] were used as a guide for choosing an appropriate exercise or activity for each session. At the beginning of each new weekly intervention group, the group would discuss exercise options. The intent of this discussion was to introduce the participants to exercise that could easily be incorporated into their daily lives, like walking, swimming, or team sport. Each group chose a different activity. Some, for example, chose walking, while others chose to go to the local gym; however, in one particular group, participants expressed anxiety about exercising in public, feeling self-conscious and anxious about their weight as well as ideas of paranoia about public places. This led the researcher to investigate options that would be appropriate for group members who had anxiety about exercising in public. A walking exercise DVD was found that enabled participants to participate in the recommended 30 minutes of exercise while taking into account their personal ability and mental health issues such as agoraphobia and paranoia. For some participants, this public aspect of the intervention was extremely problematic. For example, one participant with agoraphobia expressed concern about leaving her house so we had to tailor an activity program that suited her specific needs.

### 3.4. Issues in following the Recommended Eating Plan and Making Healthy Food Choices

The healthy eating booklet given to all participants as part of the intervention had a section where participants were encouraged to record their daily food intake. Issues around participant use of this section of the booklet included literacy. A few participants identified early in the study their personal issues with writing and reading. This was overcome by seeking support in reading and writing the weekly program from family and case managers. Other issues in following a healthy eating plan identified by participants included limited access to healthy food due to limited availability of this at their local supermarket or because of lack of affordability. The issue of affordability and access was often discussed at the weekly intervention sessions and examples and suggestions of affordable and seasonal foods were shared by the facilitator and other participants. However, many participants relied on a welfare pension, and the fact remains that living on the disability pension has a significant impact on the ability to choose healthy foods (e.g., fresh food) as they are often more expensive than takeaway or packaged foods.

A further issue was how participants understood and interpreted recommended food serving sizes and how much healthy food to eat. Food serving sizes were covered in the educational booklet provided in the intervention, as were examples of healthy food snacks [19]. One participant, for example, found a low fat ice cream that was a healthy snack; however, this participant (unlike many others) had the funds to buy many ice creams and ate excessive amounts of the low fat ice cream, up to 10 per day. This led to a significant weight gain.

### 3.5. Dual Identities: Mental Health Nurse and Researcher

In this final section, the focus is on the issues faced by the researcher. The researcher (first author) who administered the weekly intervention is a mental health nurse. While this professional identity provided her with the knowledge and experience to perform the group intervention and to work effectively with mental health consumers, it also led to a potential issue of bias due to “the therapeutic alliance.” This refers to the relationship and rapport built between nurse and the person with mental illness. The therapeutic alliance is considered an essential component of mental health nursing, and, as such, in order to effectively conduct the weekly group sessions, an alliance between participants and the researcher was developed. The partnership within the therapeutic alliance facilitates the achievement of individual health goals within a supportive and authentic relationship [20].

The weekly intervention group sessions in the study were underpinned by the spirit and philosophy of motivational interviewing (MI) as MI has been shown to be an effective management strategy to help people change risky health behaviours [21]. The spirit of MI includes concepts familiar to mental health nursing such as collaboration, evocation, and autonomy, thus enabling participants and the nurse researcher to develop authentic relationships. However, in doing so, there was the potential for blurring of the nurse nurse-researcher role and this raised tensions for the researcher with regard to balancing the research objectives with that of the therapeutic benefits.

RCTs require the researcher to take a position that is distant and focused on measurement using instruments or tools, as the objective of the experimental research is to search for truth in an objective and controlled manner [4]. Evidence indicates, however, that formation of a therapeutic alliance between the researcher and participant has the potential to influence the outcome of the research in the desired direction [16]. Therefore, the therapeutic alliance needs to be addressed in a RCT of a therapeutic intervention.

Many authors have discussed the challenges of the dual role of nurse and nurse researcher, with Chesney [22] suggesting that while a conundrum exists between the nurse nurse-researcher role, the concern is more about the researcher recognising the self and the impact this might have on the research. To extend this concept, in this study, it was the nurse's therapeutic use of self in developing a therapeutic alliance that potentially could impact the study outcomes.

The concept of reflexivity is well recognised and applied in qualitative research. Reflexivity includes self-conscious awareness of the researcher's use of self and the impact of this on the research. Being reflexive involves the researchers' acknowledgement of the value and beliefs they bring to the study and how these may affect the conduct and/or interpretation of the study findings [23]. Quantitative research, where the researcher is expected to collect data using measurement tools that reduce answers to numbers analysed statistically to produce research findings [8], does not traditionally attend to reflexivity.

In the current study, researcher awareness of their therapeutic use of self and the dual role and tension of nurse-nurse researcher are highlighted in this brief journal extract:* I often found during the groups that I was asked by participants to draw on knowledge from areas of my nursing. I would be asked for advice such as …What do you think this rash is? What should I do about the flu? Should I get this mole checked? Should I tell my case manager about the voices? Why do I have to take antipsychotic medication? *Colbourne and Sque [24] had a similar experience, where despite careful explanation of the researcher role, participants continued to request and expect “nursing” care. This example highlights the need for attention by the nurse nurse-researcher to consider the options and/or consequences of developing relationships that are distant or developing relationships that involve authentic partnerships of sharing and self-disclosure [24].

## 4. Discussion

Conducting behavioural change research with “real people” can be challenging, particularly with vulnerable groups such as those with mental illness, where the human connection between researcher and participant is an essential part of the study intervention. While attending to the important methodological elements of RCTs, the researchers in this project identified and encountered a variety of issues.

The researchers in this project, while attending to the important elements of RCTs, also identified that a variety of issues can be encountered when conducting behavioural change research with “real people,” particularly vulnerable groups such as those with mental illness, where the human connection between researcher and participant is an essential part of the research process.

While random allocation was successful as a RCT process, the desire of some participants to be involved in groups despite their random allocation needs to be considered. The human connection and the opportunity to be involved in a group where you feel you* fit in* are likely to have impacted the choice of participants to turn up each week. Participants while being encouraged to participate according to their random allocation were not turned away from weekly group sessions. The spirit of MI—collaboration, evocation, and autonomy—allows individuals to progress at their own pace [21] and was likely influential in attracting and keeping the participants attending the weekly group sessions. Group participation is often reliant on the participants of a group feeling comfortable and it is likely that the participants in this study felt comfortable with their peers. As a result of this study, a further project has been developed to investigate the experience of group participation and to gain an insight into the experience of attending healthy lifestyle groups.

As experienced in this study, recognising the impact of economics, environment, and health, both physical and mental, on nutritional choices and activity can be a challenge for clinical researchers. The reality for many people with mental illness is that living on a disability pension limits the choices available, in relation to not only nutrition but also physical activity. An important factor during this study was allowing the participants to choose activities that could be continued after the study; hence, most participants chose walking as it was convenient and did not have any associated costs. The impact of economics on nutritional choice is a reality that is challenging to resolve but nevertheless needs to be highlighted as a potential limitation for participants in a healthy lifestyle program.

The use of a reflexive approach as described by Finlay [25], which encompasses continual evaluation of subjective responses and research method, was essential for the healthy lifestyle intervention in this study to proceed. If the typical objective researcher stance had prevailed during the data collection phase of the project (group intervention), participants may not have engaged for the full 12-week intervention and the role tension between “nurse” and “nurse researcher” may not have arisen. Johnson and Clarke [26] describe such an experience as “stepping out of the researcher role” and suggest that the pressures to do so often occur when the participant knows that the researcher is a nurse. Further, to this perception of external pressure to cross the conventional researcher/participant barrier, is the internal socialisation of nurses to provide care. However, we agree with Colbourne and Sque [24] despite attempting to remove the “nurse” from the nurse-researcher, “if the nurse cannot be removed from the researcher then why pretend otherwise” (p. 303).

The use of a reflexive approach in quantitative studies could influence data collection and participant involvement in a number of ways, particularly in sensitive research where the researcher has contact with vulnerable people. Johnson and Clarke [26] suggest that, in terms of the researcher-participant relationship, a reflexive approach, enabling the researcher to “step out” of the researcher role, may facilitate increased access to participants and data, but they caution the researcher to be alert for coercion and exploitation. Carolan [27] recognises the increasing credibility that a reflexive approach can bring to qualitative studies. We contend that it is possible that a reflective approach could offer similar benefits for quantitative studies when researchers are delivering a behavioural intervention. We suggest that, contrary to views that reflexivity could contaminate the research process in quantitative research, these studies can be enriched by the reflexive process through disciplined self-awareness and self-management by the researcher. Paradoxically, rather than increasing bias, this process may assist in reducing subjectivity and enabling further objectivity on the part of the researcher. The researcher's objective stance can be maintained by the use of reflective practice to bring into focus what may be influencing the data collection of a project. In this study, without acknowledging the impact of mental illness on participants' involvement in the project, the research findings may not have been an accurate reflection of causal relationships. In this case, the use of reflexivity and surfacing the researcher's beliefs and practices have led to a more objective stance—acknowledging the impact of mental illness on participation in the research—that has decreased the potential bias of the study.

## 5. Conclusion

The challenges experienced by the research team in this study include understanding the issue of dual identity for the nurse-nurse researcher and recognising the impact of economics, environment, and health, both physical and mental, on participants' nutritional choices and activity in this healthy lifestyle intervention. While the influence of reflexivity in RCTs with vulnerable groups has been recognised in this paper, the authors have also identified the importance of the participants' experiences and stories. The use of reflexivity enables the researcher to remain engaged with the research population while also enabling the researcher to maintain an objective stance. By deconstructing the experience of a RCT, the researchers have been able to identify the challenges that can be planned for future behavioural interventions. The challenges for future researchers include recognising and planning for reflexivity in qualitative and quantitative research and acknowledging the importance of the nurse in the nurse researcher role. Future research on behavioural interventions will be enriched by appreciation and inclusion of the person's story as part of the intervention.
